# Kimchi Lactic Acid Bacteria Starter Culture: Impact on Fermented Malt Beverage Volatile Profile, Sensory Analysis, and Physicochemical Traits

**DOI:** 10.4014/jmb.2403.03011

**Published:** 2024-06-28

**Authors:** Moeun Lee, Daun Kim, Ki Won Lee, Ji Yoon Chang

**Affiliations:** 1 Fermentation regulation research group, World Institute of Kimchi, Gwangju 61755, Republic of Korea; 2 Biomodulation Major, Department of Agricultural Biotechnology, Seoul National University, Seoul, 08826, Republic of Korea; 3 Department of Food and Nutrition, Chosun University, Gwangju 61452, Republic of Korea; 4 Advanced Institutes of Convergence Technology, Seoul National University, Suwon 16229, Republic of Korea; 5 Division of Applied Life Science (BK21), Graduate School, Gyeongsang National University, Jinju, Gyeongnam 52828, Republic of Korea; 6 Institute of Smart Farm Research Center, Gyeongsang National University, Jinju, Gyeongnam 52828, Republic of Korea

**Keywords:** *Levilactobacillus brevis* WiKim0194, kimchi starter, fermented malt beverage, sensory evaluation, electronic tongue, electronic nose

## Abstract

Starter cultures used during the fermentation of malt wort can increase the sensory characteristics of the resulting beverages. This study aimed to explore the aroma composition and flavor recognition of malt wort beverages fermented with lactic acid bacteria (*Levilactobacillus brevis* WiKim0194) isolated from kimchi, using metabolomic profiling and electronic tongue and nose technologies. Four sugars and five organic acids were detected using high-performance liquid chromatography, with maltose and lactic acid present in the highest amounts. Additionally, e-tongue measurements showed a significant increase in the sourness (AHS), sweetness (ANS), and umami (NMS) sensors, whereas bitterness (SCS) significantly decreased. Furthermore, 20 key aroma compounds were identified using gas chromatography-mass spectrometry and 15 key aroma flavors were detected using an electronic nose. Vanillin, citronellol, and β-damascenone exhibited significant differences in the flavor profile of the beverage fermented by WiKim0194, which correlated with floral, fruity, and sweet notes. Therefore, we suggest that an appropriate starter culture can improve sensory characteristics and predict flavor development in malt wort beverages.

## Introduction

Cereal-based beverages crafted through lactic acid fermentation are attracting considerable attention due to their nutritional content and functional attributes, offering qualities such as low cholesterol, non-alcoholic, gluten-free, and functional properties [1]. These beverages hold potential as substrates for the development of synbiotic drinks, combining probiotics and prebiotics to synergistically promote gut health. Moreover, they function as readily accessible and cost-effective sources of energy, containing abundant prebiotic ingredients like dietary fibers and resistant starch, which efficiently facilitate microbial growth in the presence of moisture and hydrolytic enzymes [2]. However, as consumers place greater emphasis on flavor enhancement, products must prioritize appealing taste and aroma, along with nutritional quality, to be selected in the market [3].

Wheat and barley are the commonly used grains in the brewery industry. Wheat and barely contain many nutrients, including proteins and bioactive compounds. Following malting and mashing, which are necessary for wort production, fermentation by lactic acid bacteria (LAB) enhances nutrient availability and contributes to the development of attractive flavors [4]. Prior to yeast fermentation, malting and lactic acid fermentation serve two purposes: enzymatic hydrolysis of the grain structure and enhanced flavor formation, potentially impacting the final beverage flavor [5]. In 2022, South Korea was the 17th largest beer consumer, and accounted for USD 3,716 million in sales [6]. In recent years, consumers are turning to craft beer over mass-produced brands. Craft beer is typically produced by small, independent breweries that use traditional brewing methods and focus on creating beers with unique flavor and quality [7]. Hence, optimal malting and mashing conditions are necessary for the production of lactic acid-based beverages using various cereals.

LAB are prevalent in nature and widely employed as food-grade microorganisms in diverse fermentation industries. There is growing interest in the industrial applications of LAB, particularly their application as starter cultures; however, the quality of fermented products depends on the specific LAB species used in the fermentation process [8]. The flavor profiles are affected by different LAB fermentation species. For example, *Lactobacillus* and *Pediococcus* are commonly associated with lactic acid production in sour beer [9]. LAB are considered important for active metabolites beyond lactic acid. These metabolites include various organic acids such as acetic and formic acid, esters, aldehydes, and ketones, along with phenolic and non-phenolic compounds [10]. In addition, LAB species *Lactobacillus brevis* (*L. brevis*), *Lactobacillus Plantanum* (*L. plantanum*), and *Lactobacillus curvatus* (*L. curvatus*), that are commonly found in beer, have been identified in beer [11]. Aroma composition and sensory acceptance have been reported for strains currently used in breweries (*e.g.*, *L. amylolyticus*, *L. plantarum*) or in malt wort (*e.g.*, *L. brevis*) [12]. Studies on LAB strains from various origins are necessary to enhance flavor and improve the characteristics of malt-based beverage production.

Kimchi, a traditional fermented food in Korea, is fermented using various LAB types that produce a range of metabolites, including lactic acid and acetic acid (imparting a sour taste), mannitol (providing cool sweetness), and acetoin (contributing to a unique fermentation aroma) [13]. The optimal fermentation by specific LAB starters results in the formation of a diverse array of metabolites, contributing to the complex sensory properties of final products, including a savory taste [14]. In this study, we aimed to characterize the growth properties of various LAB species isolated from kimchi and applied them to wort fermentation. Our focus was on characterizing their growth properties and evaluating the presence of metabolites that affect flavor and sensory properties.

## Materials and Methods

### Wort Preparation and Starter Culture

A wort with a concentration of 12% was made by mixing 80% standardized barley malt with distilled water. Next, the mixture was autoclaved at 110°C for 10 min. After cooling, the debris was removed. Unhopped malt, employed as a base in the brewing experiment, was used to propagate and prepare the LAB starter culture, as well as for the microbial growth experiment. Eight previously identified and selected kimchi LAB strains were used in this study (Table 1). The isolated strains were cultured in de Man, Rogosa, and Sharpe (MRS) medium (BD Difco, USA) at 30°C for 24 h. The LAB strains were activated in MRS broth for 24 h and pre-cultured in wort for an additional 24 h at 30°C prior to the experiment. Subsequently, the cells were pelleted at 6,000 ×*g*, and 4°C for 10 min, and washed twice with sterile PBS. With each LAB strain, fermentation was conducted at laboratory scale under static conditions for 72 h at an inoculation rate of 1% (v/v). The fermented beverages were immediately stored at -20°C for sensory evaluation and at -80°C for aroma compound analysis.

### Microbial Growth

The growth experiments were conducted in 96-well microtiter plates, with each well containing a total volume of 200 μl. The strains were individually inoculated at approximately 6.0 log10 CFU ml^−1^ in both MRS broth and wort, and then incubated at 30°C for 42 h. To prevent evaporation, a sterile transparent film was used to cover the surfaces of the wells. Control samples (non-inoculated media) and each experimental trial were set up in triplicate and monitored using a microplate reader (Safire, Tecan, Switzerland) to measure optical density (OD) at 600 nm every 3 h. Prior to reading, the microtiter plate was shaken for 10 sec.

### Chemical Analyses

pH of the LAB-fermented wort was determined using a pH meter (Orion 3-Star; Thermo Fisher Scientific, USA). Next, the samples were titrated with 0.1 N NaOH to achieve a pH of 8.3, facilitating the assessment of the titratable total acidity. Total acidity was calculated using a predefined equation [15]. Additionally, the total soluble solid content, measured as the °Brix value, was assessed for each sample using a digital refractometer (model WM-7, Atago Co., Ltd, Japan).

### High-Performance Liquid Chromatography

The level of organic acids and sugars in the samples was assessed during the fermentation process using high-performance liquid chromatography (HPLC; Waters Alliance e2695; USA), as previously established protocols [16]. Quantitative analysis of target compounds was performed using standard curves.

### Electronic Tongue Analysis

Tase assessments of both fermented and non-fermented worts were conducted using electronic tongue analysis. This analysis employed an α-Astree II electronic tongue system (Alpha MOS, Toulouse, France), which featured a sensor array consisting of seven chemical sensors: AHS (sourness), NMS (umami), CTS (saltiness), ANS (sweetness), SCS (bitterness), and PKS, and CPS (comprehensive taste), in addition to an Ag/AgCl reference electrode. All tests were performed in triplicate.

### Electronic Nose Analysis

Volatile compounds in both fermented and non-fermented wort were assessed using a rapid gas chromatography electronic nose system (Heracles II, Alpha M.O.S., France), as previously described [17], with minor adjustments. Two columns of different polarities were employed: a nonpolar MXT-5 (5% diphenyl) column and a moderately polar MXT-1701 (14% cyanopropylphenyl) column, each measuring 10 m in length and 180 μm×0.4 μm. To extract volatile compounds from the sample matrix, vials were incubated with continuous agitation (500 rpm) at 50°C for 20 min in a controlled thermostatic agitator. After incubation, 5 ml of the headspace was extracted using a syringe at 60°C and introduced into the GC system at 200°C for 45 sec at a flow rate of 125 μl/s, with hydrogen N7.0 as the carrier gas. Flame ionization detectors (FIDs) were maintained at a temperature of 260°C. Each test was repeated five times.

### Statistical Analysis

Principal component analysis (PCA) was conducted using the "prcomp" function within the "ggfortify" package of R software (v3.3.2; available at https://www.r-project.org/). Statistical analysis involved using a Student's t-test (utilized in PRISM) to compare groups. Results with p-values below 0.05 were deemed statistically significant.

## Results and Discussion

### Strain Selection

**Fig. 1:**
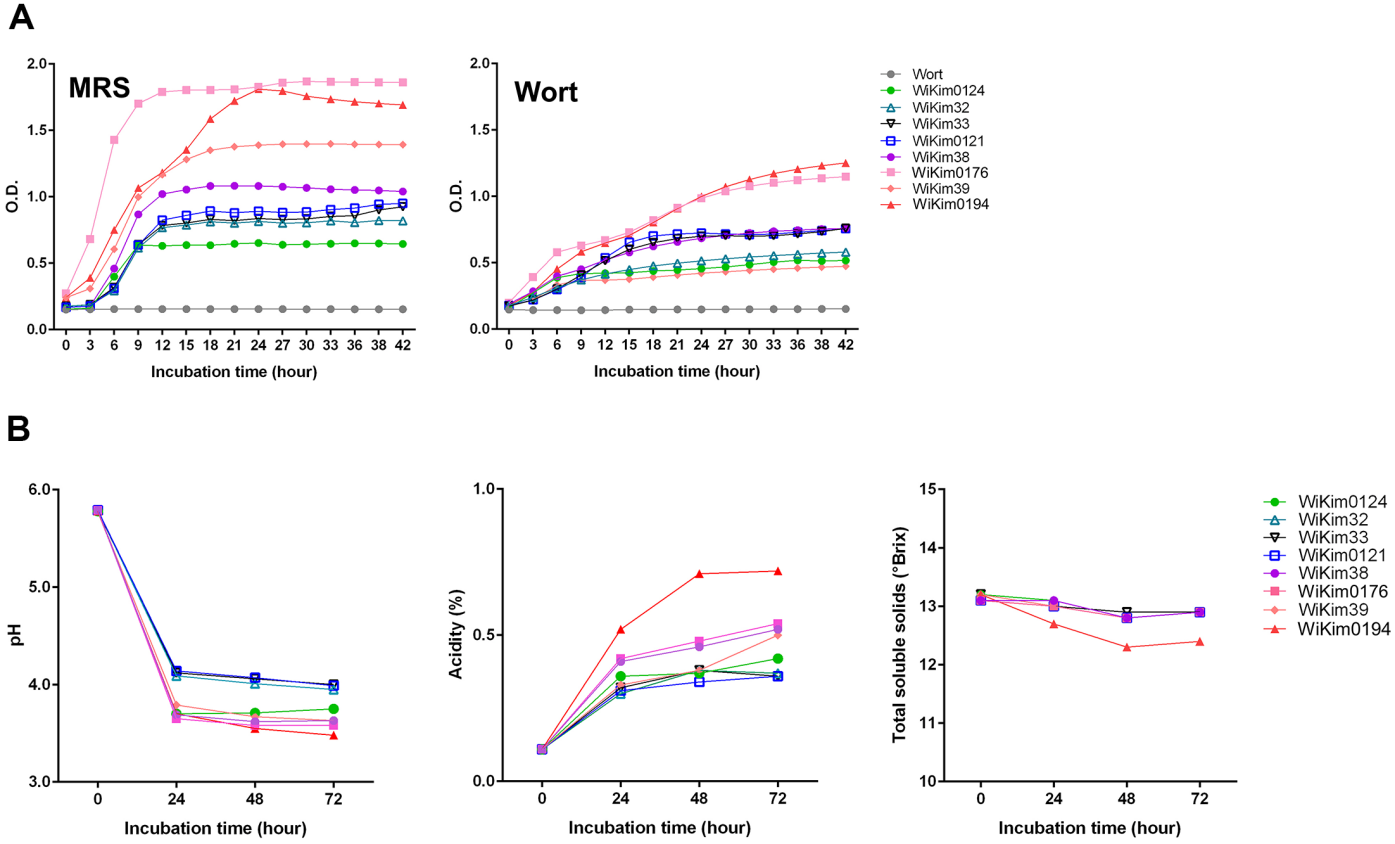
Properties of malt wort beverages during fermentation using eight LAB strains. (**A**) Growth curves of the eight LAB strains. (**B**) pH profiles, titratable acidity, and total soluble solids. All experiments were conducted in triplicate.

Growth kinetics, acidification rate, sugar utilization, and acid production were analyzed to determine the most suitable bacterial starters. Out of the eight isolates, WiKim0176 and WiKim0194 showed the highest optical density (OD) throughout the entire incubation period in MRS broth. These strains also exhibited the most rapid growth in malt wort at the end of the incubation period, with WiKim0194 reaching its maximum OD (Fig. 1A). In contrast, the remaining isolates demonstrated very weak growth compared to MRS broth. The pH of each culture was measured every 24 h. As a general trend, a fast drop was observed within the first 24 h of fermentation, followed by a mild decline thereafter. Similar pH values were observed until 72 h with the exception of *Leuconostoc* spp.. However, the highest acidification rate was observed with the WiKim0194 strain, throughout the malt wort fermentation process. Concurrently, there was a noticeable reduction in the sugar content of the total soluble solids (Fig. 1B). The degradation of carbohydrates in malt wort during fermentation leads to the generation of organic acids that influence its flavor profile and contribute to pH fluctuations [18]. Screening for strains demonstrating adequate acidification in malt wort led to the selection of WiKim0194. Acidification of malt wort by LAB is strain-dependent. Various fermentation types and metabolic characteristics (*e.g.*, carbohydrates and proteins) of the strains may account for these results [19].

### Changes in Sugar and Organic Acid Concentrations throughout Fermentation

**Fig. 2:**
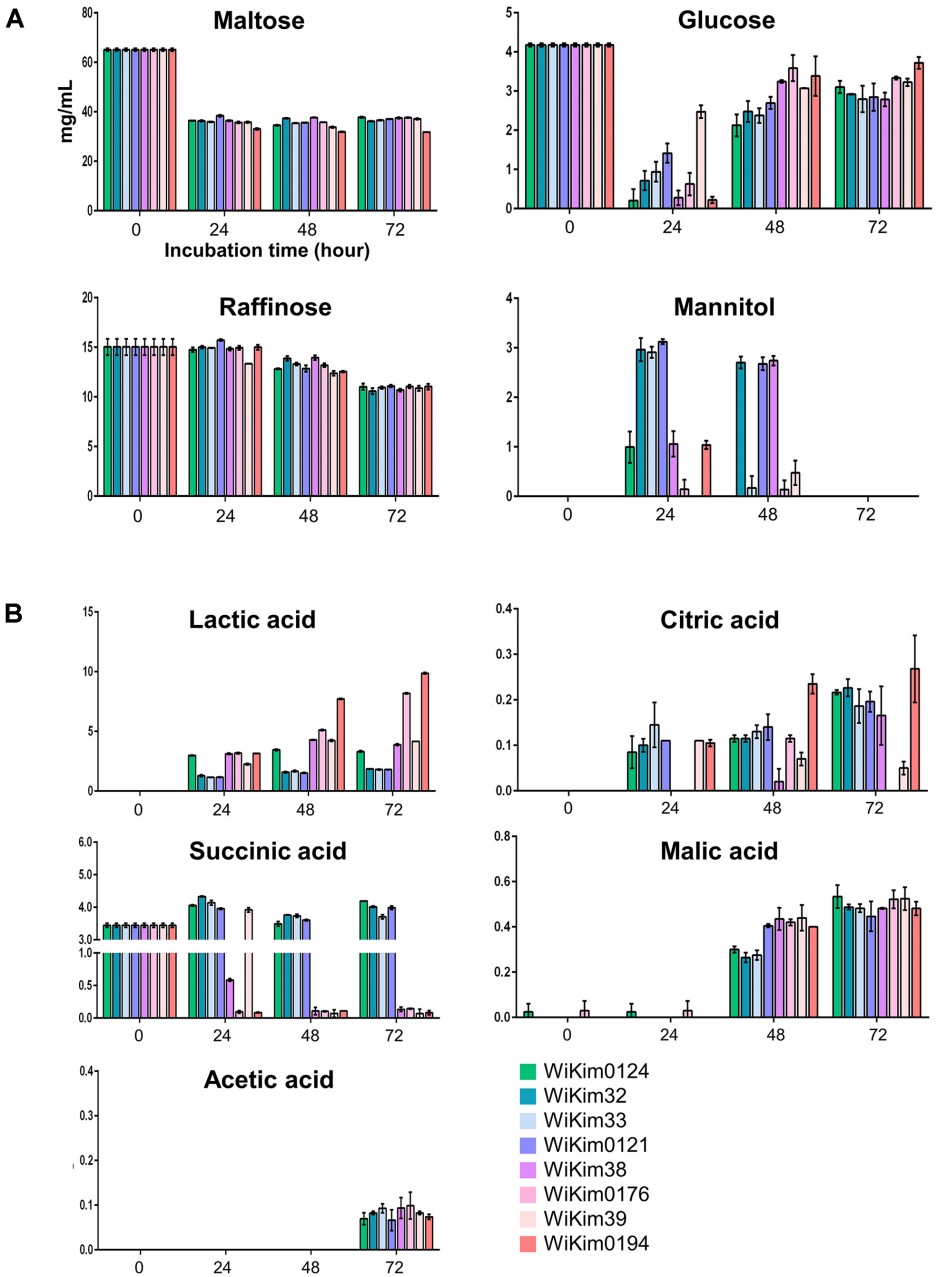
Metabolomic profiles of malt wort beverages. (**A**) Sugar content. (**B**) Organic acid contents. All experiments were conducted in triplicate.

Fig. 2 illustrates the changes in the concentrations of sugars (maltose, glucose, raffinose, and mannitol) and organic acids (lactic, citric, succinic, malic, and acetic acids) during malt wort fermentation with different starter cultures. As shown in Fig. 2A, raffinose concentrations declined steadily until 72 h, with no significant variation between the strains. Maltose was the primary fermentable sugar in the malt wort, with other sugars making up a minor portion. During the early stages of growth, exhibited robust maltose utilization. Glucose levels declined by the 24-h mark, indicating consumption, until it became undetectable. Subsequently, the glucose levels in sour wort increased with the use of all LAB strains, with variations among strains. Particularly the WiKim0194 strain showed highest increase. This increase in glucose production could be linked to the ability of the LAB strains to hydrolyze starch. A previous study reported the ability of LAB to break down sorghum starch, leading to an acidified end product, notably lactic acid, which lowers pH [20]. Their metabolic activity in metabolizing maltose into glucose enhances carbohydrate utilization capacity, influencing the microbial ecosystem [21]. The production of organic acids is a result of sugar metabolism [22]. Lactic acid, citric acid, succinic acid, malic acid, and acetic acid are typical non-volatile acids resulting from LAB-fermented foods and beverages [23]. Fermentation can produce significant amounts of lactic acid, which serves as an excellent flavoring agent. Its moderate sourness lends a unique taste and has an appetite-enhancing effect [24]. During sour wort production, lactic acid was the predominant compound produced (Fig. 2B). WiKim0194-fermented wort exhibited the highest lactic acid production (9.86 mg/ml at 72 h), followed by WiKim0176 (8.19 mg/ml at 72 h), which is consistent with the physicochemical findings. The citric, malic, and acetic acid levels increased in fermentations with all starters. Succinic acid, initially at 3.40 mg/ml, declined to undetectable level post-fermentation with WiKim0176, WiKim39, and WiKim0194. Succinic acid can serve as a metabolic intermediate utilized or produced during fermentation. While it is typically formed by yeast, its presence tends to decrease in beverages undergoing LAB fermentation [25]. Acetic acid, while slightly irritating, can soften food when found in low concentrations; hence, a moderate level of acetic acid can enhance the sourness of food [26]. However, in this study, acetic acid was present in fermentations with all starter strains at 72 h, without notable differences between strains. Based on these results, the taste and aroma characteristics were further analyzed in the WiKim0194 starter, which exhibited the most rapid and effective fermentation.

### Sensory Characteristics of Fermented Malt Beverage

**Fig. 3:**
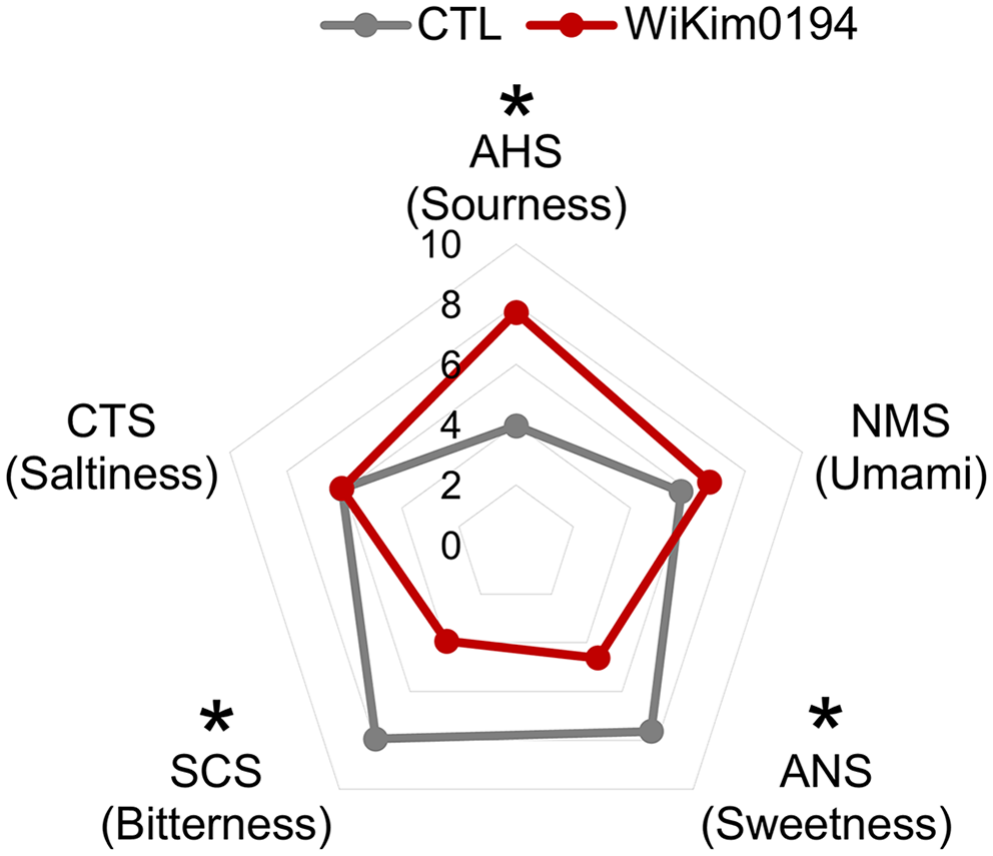
Sensory attribute profiles of WiKim0194-fermented malt wort constructed by e-tongue. SCS, bitterness; CPS, comprehensive taste; CTS, saltiness; NMS, umami; ANS, sweetness; AHS, sourness. Statistical significance was analyzed by Student's *t*-test (*p < 0.05). All experiments were conducted in triplicate.

**E-tongue analysis.** The E-tongue is an electronic equipment that converts electrical signals into taste perception, facilitating an unbiased evaluation of food taste. It has high sensitivity and eliminates subjective elements inherent in conventional sensory assessments [27]. Fig. 3 illustrates the response values of the sensory characteristics of the fermented malt beverage, including sourness (AHS), bitterness (SCS), saltiness (CTS), umami (NMS), and sweetness (ANS), as obtained from E-tongue analysis. Significant changes in signal values were detected during the fermentation of malt wort, and the major sensory difference detected was an increase in sourness. This is likely due to lactic acid fermentation, in which lactic acid is the primary product of WiKim0194. The second major sensory difference was a decrease in bitterness. The significant decrease in perceived bitterness was associated with the generation of other intensely flavored compounds that enhance taste attributes. These compounds may help obscure the unsavory bitter taste, with organic acids, including lactic acid, detected at higher levels in the fermented malt wort, possibly contributing to this effect [28]. Sweetness decreased by 0.6-fold, likely contributing to the carbohydrate metabolism of WiKim0194, which is consistent with the decrease in sugar content. Sugars and organic acids play a role in flavor formation. However, various secondary metabolites like amino acids, flavonoids, and alkaloids are also essential contributors to flavor [29]. Given that flavor perception is collectively determined by sugars, acids, and other metabolites, which can undergo changes and alterations within and among these chemicals [30], further study is necessary to employ omics-based techniques like metabolomics. This will enable a more precise identification of flavor-related chemical components that might impact e-tongue analysis.

**Fig. 4:**
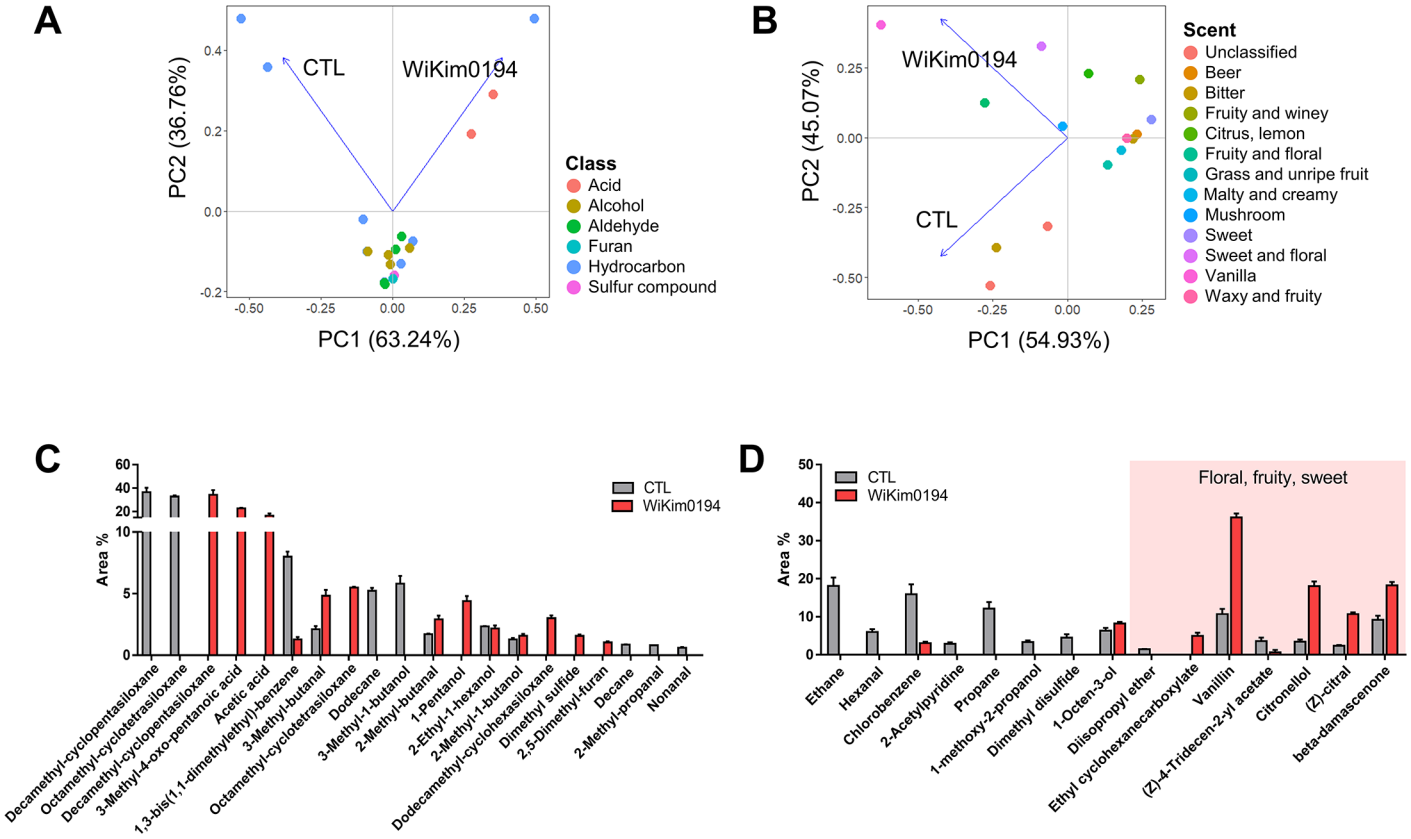
Comparison of aroma compoundse-nose signal values in WiKim0194-fermented malt wort. (**A**) The PCA scores constructed based on the composition of aroma compounds obtained from GC-MS. (**B**) The PCA scores constructed based on the composition of e-nose signal value profiles. (**C**) Key fermentative aroma compounds in fermented malt wort obtained from GC-MS. (**D**) Key fermentative aroma compounds based on e-nose signal values. All experiments were conducted in triplicate.

**Key aroma compounds.** The comprehensive flavor profile is influenced by the distinct contributions of each essential aromatic compound. To identify the key odor-active compounds that contribute the most to the final flavor of the fermented malt wort, GC-MS and an E-nose system were utilized. The principal component analysis (PCA) plot explains the correlation between each beverage produced by fermentation, the corresponding aroma compounds (Fig. 4A), and sensory descriptors (Fig. 4B). The plot of the GC-MS profiles revealed a 63.24%variation in the sample set, enabling the identification of two sample groups and characteristic aroma compounds. The beverages fermented with WiKim0194 were distinctly different from each other and from those fermented by CTL. Large variations were observed among the aromatic compounds in the hydrocarbon and acid categories. Meanwhile, the plot of the e-nose profiles revealed a 45.07% variation in the sample set. Beverages fermented with WiKim0194 exhibited relatively attractive flavor notes such as vanilla, fruity, floral, citrus, lemon, and sweet. However, beverages from the CTL group had relatively less attractive flavor notes, such as bitter or unclassified. This observation is consistent with the relatively high levels of key aroma compounds recorded during the GC–MS analysis (Fig. 4C). Acetic acid accounted for 16.54% of the variation in WiKim0194-fermented malt wort, as indicated by GC-MS results. Recognized for its sourness and vinegar-like flavors, acetic acid significantly affects the sensory characteristics. Additionally, the quality of the 'sour odor,’ distinct from acidic taste, often presents as a refreshing and well-balanced scent [31]. The increase in fruity and floral aromas was probably due to the generation of intense aromatic compounds during lactic acid fermentation [28]. Several unidentified compounds (decamethylcyclopentasiloxane, 3-methyl-4-oxo-pentanoic acid, and octamethyl-cyclotetrasiloxane) were detected, along with potential fruity and floral aromatic compounds, such as 3-methyl-butanal [32] and 1-pentanol [33]. This is also consistent with the fact that the positive flavor attributes in WiKim0194-fermented malt wort were ethyl cyclohexanecarboxylate [34], vanillin [35], citronellol [36], (Z)-citral [37], and beta-damascenone [38], with floral, fruity, and sweet flavor notes, respectively, recorded during e-nose analysis (Fig. 4D). The bitter odor and off-flavor of WiKim0194-fermented malt wort are thought to have decreased due to a reduction in the compounds responsible for the bitter flavor, such as chlorobenzene [39] and 2-acetylpyridine [40].

Our results indicate that the addition of a specific starter significantly affects the sensory properties of LAB-fermented malt wort beverages, particularly in terms of sourness, bitterness, and sweetness perception, as revealed by e-nose and e-tongue analyses. Additionally, our findings offer valuable insights into the multitude of metabolites that affect the flavor, fragrance, and overall sensory perception of these beverages. These insights enrich our understanding of LAB starters in sour beer production.

## Figures and Tables

**Table 1:** Lactic acid bacteria strains used in this study.

Strain	Gene bank ac no.	Code
*Lactococcus lactis* WiKim0124	MZ424472.1	WiKim0124
*Leuconostoc mesenteroides* WiKim32	NZ_CP037752.1	WiKim32
*Leuconostoc mesenteroides* WiKim33	CP021491.1	WiKim33
*Leuconostoc mesenteroides* WiKim0121	CP098784.1	WiKim0121
*Latilactobacillus curvatus* WiKim38	KU936208.1	WiKim38
*Latilactobacillus sakei* WiKim0176	-	WiKim0176
*Latilactobacillus sakei* WiKim34	OL638252.1	WiKim34
*Companilactobacillus allii* WiKim39	NR_159087.1	WiKim39
*Levilactobacillus brevis* WiKim0194	-	WiKim0194
